# Physical Properties of an Eco-Sustainable, Form-Stable Phase Change Material Included in Aerial-Lime-Based Mortar Intended for Different Climates

**DOI:** 10.3390/ma15031192

**Published:** 2022-02-04

**Authors:** Antonella Sarcinella, José Luís Barroso de Aguiar, Mariaenrica Frigione

**Affiliations:** 1 Department of Innovation Engineering, University of Salento, 73100 Lecce, Italy; antonella.sarcinella@unisalento.it; 2 Department of Civil Engineering, University of Minho, 4800-058 Guimarães, Portugal; aguiar@civil.uminho.pt

**Keywords:** aerial lime mortars, circular economy, energy efficiency, phase change materials (PCMs), thermal energy storage (TES)

## Abstract

The aim of this experimental investigation was to produce a form-stable phase change material (PCM) able to reduce the need for nonrenewable energy resources required for the heating/cooling of buildings located in regions characterized by different climatic conditions. The innovative PCM must also be sustainable and must be produced according to the principles of the circular economy. To achieve such ambitious goals, a form-stable, sustainable PCM was produced through vacuum impregnation. The form-stable PCM was produced starting from a low-toxicity, low-flammability polyethylene glycol of medium molecular weight (PEG 800), which was included in porous stone granules obtained as waste products of the cutting/processing of local (Lecce) stone. The thermal properties and thermal stability of PEG 800 and of its PCM-composite were evaluated by employing differential scanning calorimetry (DSC) and thermo-gravimetric analysis (TGA). The appropriate parameters to perform the impregnation procedure were identified through rheological and calorimetric analyses. A simple leakage test was performed to assess if the PEG polymer can leak from the stone flakes. Finally, the new PCM was added as an aggregate in aerial-lime-based mortars, and the mortar’s properties were analyzed in fresh (workability) and hardened (flexural and compressive strength and thermal characteristics) states for potential applications, particularly in ancient buildings.

## 1. Introduction

The world is increasingly concerned about excess energy consumption leading to serious consequences for the environment. Among all sectors, construction is one of the biggest contributors to this issue. This has been confirmed by the International Energy Agency (IEA) [1]. The main cause for this high energy utilization seems to be the increasing demand for heating and cooling necessary for human thermal comfort. Because of this increasing habit of using more and more cooling and heating devices, public and political authorities are trying to adopt new policies and new strategies to promote energy efficiency in buildings, thus reducing energy consumption. In addition, the academic community is also highly motivated to find new methods and alternative technologies to reduce global energy consumption. These two realities are on the same path, and their collaboration could promote an ever-lower dependence of buildings on fossil fuels. 

Interest in new materials capable of improving energy efficiency is growing steadily, and a very attractive and well-consolidated approach seems to be thermal energy storage (TES) [2,3], with latent heat thermal energy storage (LHTES) one of the available methods. In this approach, a phase change material (PCM—a system able to absorb and release energy due to a change in the external temperature modifying its physical state) is used [4]. A PCM transitions between liquid and solid states within the environmental temperature range; during this transition, it stores/releases energy [5,6,7]. Thus, the integration of a PCM into a building component, such as mortar, can reduce indoor temperature variations, improving human comfort with a reduced consumption of energy [8,9,10] and an increase in the use of renewable energy. Several experimental investigations have been recently conducted in order to examine the behavior of a PCM integrated into construction materials; its inclusion in mortars used on the interior walls appears to be the most advantageous solution [11,12]. There are several methods for including the PCM into mortar—according to the current literature, the form-stable method is the most promising [13,14] and is also able to guarantee good thermal stability [15]. 

The “form-stable composite PCM” formula only refers to a system composed of an inert porous matrix that can absorb an optimal amount of PCM. However, it is important that when the temperature reaches the melting point of the PCM it does not leak from the matrix [16]. This feature can also extend the life of the material by preventing its degradation during thermal cycling. This is an obvious advantage for installations that require long-term performance, such as in the construction industry [17]. 

One of the most attractive aspects of the form-stable method is its cheapness and simplicity in implementation. Very simple and easily adaptable equipment is necessary for its application, allowing the obtainment of a final material with the required features [15,18]. With an appropriate selection of raw constituent materials, a sustainable composite PCM can be produced and used as aggregate in different mortar formulations [16,19,20,21].

This work is one part of larger research, the intent of which is to produce a form-stable material to improve the energy efficiency of buildings. This material should be able to reduce the need for nonrenewable energy resources for heating/cooling, with a consequent reduction of CO_2_ emissions. The form-stable material should also be sustainable and should respect the principles of the circular economy. To meet these criteria, polyethylene glycol (PEG) was selected as the polymerically active component for the PCM due to its sustainable characteristics, favorable properties, and inexpensiveness. The exploitation of waste materials from stone processing as the inert support for the form-stable PCM, furthermore, respects the principles of the circular economy. The valorization of waste materials for the production of new products, in fact, is in line with the principles of sustainability and allows a reduction of costs for their disposal. PEG polymer has been adapted to many different applications; thanks to its availability in different molecular weights, it is characterized by different melting temperatures. These features make PEG a very versatile sustainable material [22].

Several studies reported the production of form-stable PCMs based on PEG [23,24,25,26]. Among other advantages, a low leakage of PEG from the porous support was observed during its phase-change process [19,27,28], along with thermal stability and good LHTES. A form-stable PCM is generally obtained by impregnating an inert matrix, such as diatomite, acetylene black, expanded graphite, or expanded vermiculite. The melting and crystallization temperatures of the PEG-based, form-stable materials were found to be lower than those of pure PEG due to the surface interaction between PEG and the porous substrate [29,30]. With regard to the latent heat storage capacity, since the relation between PEG mass percentage in the PCM and its latent heat is linear, the enthalpy generally decreases with the reduction of the PEG content [31,32,33,34]. In most of the papers reporting the use of PEGs to produce PCMs, polyethylene glycol polymers with molecular weight not lower than 1000 have been employed. To the best of our knowledge, only a few works proposed PEGs with lower grades (e.g., PEG 800 [19,30,35] and PEG 600 [36,37]) to manufacture PCMs. However, leakage issues in the melting state limit the use of such PEGs as a form-stable PCM in real-world applications. Consequently, these form-stable PCMs have very rarely been embedded in mortar; generally, they have been thermally characterized and proposed as effective PCMs for use in different TES applications [30,35,36,37].

The incorporation of a form-stable, PEG-based PCM in a mortar formulation generally leads to reductions in mechanical properties when compared to the same mortar without PCM [31,38,39,40]. It is reported that the inclusion of any PCM in mortar negatively affects its mechanical performance [8,41,42]; therefore, gypsum and cement are the binders more often used due to their intrinsically good mechanical properties [43,44,45]. However, mortars containing PCM can be employed for interior/exterior coatings; in such applications, lower mechanical strength values are sufficient.

Aerial lime is frequently used in restoration and conservation applications of architectural heritage and historic buildings as it responds to important functional requirements, such as high chemical–physical compatibility with pre-existing substrates [46]. It was used very often as binder for mortars containing a microcapsule PCM, and their mechanical and thermal properties were investigated [45,47]; their durability has also been studied [48,49]. Therefore, our investigation turned to the study of aerial-lime-based mortars containing form-stable PCM. Historical ancient buildings are frequently rehabilitated to host cultural events; they require, therefore, some adaptative interventions. In such buildings, overly invasive interventions are not feasible, and it may be impossible to install heating or cooling systems. In such cases, an aerial-lime-based mortar containing a PCM can be proposed; in this way, it is possible to comply with the requirements imposed on such buildings while ensuring the thermal comfort of people. 

In previous research [31,50,51,52], a novel, sustainable, form-stable PCM was produced starting from a low-toxicity/low-flammability PEG polymer of medium molecular weight (PEG 1000) [50]. This polymer was easily included in waste natural material: flakes of porous stone derived from extraction and processing of the stone. The sustainable and low-cost product was added to mortars based on different binders (aerial lime, hydraulic lime, gypsum, or concrete) [31,51], which were demonstrated to be efficient in reducing indoor temperature fluctuations, especially in warm seasons and in buildings located in Mediterranean regions. It was, in fact, found that PEG 1000 does not undergo its phase change at “low” temperatures, which means during the winter season [52]. In order to extend the temperature range and make the PCM effective even at low temperatures, a new form-stable PCM was produced. Starting from the results of the previous studies, in this work a PEG polymer with a lower molecular weight (PEG 800) was selected on the basis of its capability to complete the phase transition at lower temperatures. The PEG 800 was included in waste porous-stone fragments, using the same already-successfully experimental procedure. The obtained PCM was then added as aggregate to an aerial-lime-based mortar; the properties of this mortar and its adequacy in terms of workability, compressive and flexural strengths, and thermal characteristics were then assessed.

The use of PEG 800 as a polymeric component for the PCM is expected to affect the thermal energy storage and the release capacity of the mortar at lower temperatures than previous examples [52]. In the previous study, PEG 1000 was an effective PCM in the warmer seasons in a climate typical of the Mediterranean area; therefore, PEG 800 is expected to be effective in the same Mediterranean regions but during colder seasons or in regions located in colder (continental) climates.

## 2. Materials and Methods

### 2.1. Materials

The materials used in this work are listed in Table 1. All of them have been used in previous work [31] except for PEG 800, which has been substituted for PEG 1000. As will be described in depth later, PEG 800, having a different melting/crystallization temperature than PEG 1000, is able to change the final thermal properties of the composite, eco-sustainable product.

Lecce stone (LS) and polyethylene glycol 800 (PEG 800) were chosen to create the new eco-sustainable, form-stable PCM composite, to be added to aerial lime mortar as aggregate. As found in a previous study [31], thanks to its high open porosity [53], LS is a suitable matrix where the selected polymer, at liquid state, can be easily included. The pore size distribution of Lecce stone was previously determined by mercury intrusion porosimetry (MIP), and the results were reported in [31]: most of pore sizes (61%) were between radii of 0.25 and 2 μm. The average pore radius was 0.054 ± 0.036 μm, and the total open porosity was 30.33 ± 0.99%. These porosimetric characteristics were reproducible and comparable to those previously reported in the literature [54,55].

The selection of this stone support was also driven by the possibility to exploit a local waste material: LS fragments derived from the extraction from the quarries located in the Salento area (Cursi, Lecce, Italy). These fragments were ground with a mill and then sieved in order to obtain particles with a specific granulometry suitable for their use as aggregate for the mortar. The granulometry used was in the range of 1.6 to 2.0 mm. 

PEG 800 is a thermoplastic polymer which was supplied in solid form. Due to the availability of different grades of polyethylene glycol, in this study a molecular weight of 800 was selected due to its melting temperatures range being suitable for the climatic conditions for which it was intended, i.e., around 26 °C to 32 °C, as reported on the product data sheet. 

The PCM aggregate, based on PEG 800 and LS, to be added to the mortar, was prepared through the form-stable method, as previously described [31]. With this procedure, 10 g of PEG at liquid state was absorbed by 50 g of Lecce stone granules by employing a vacuum impregnation process, whose details can be found in [31]. To obtain PEG 800 in its liquid state, it was preheated at 40 °C; this temperature was selected based on the results from rheological and thermal analyses (as specified in the next paragraph). The final material obtained through this procedure was a composite material, indicated as LS/PEG 800. Aerial lime was used as binder to produce the mortar. A control formulation was also prepared using the LS without PEG as aggregate. A superplasticizer (SP) was employed in order to limit the amount of water required. Table 2 summarizes the mortar formulations. The mortars were prepared according to European Standard EN 998-1 [56] and by taking into account the results from our previous investigations on mortars containing PCMs [31,47,49,51,57,58]. 

### 2.2. Methods

The rheological characterization of PEG 800 was carried out in order to identify the most appropriate temperature to employ in the impregnation process to achieve a high inclusion of liquid PEG 800 in the pores of the Lecce stone granules. A strain-controlled rheometer (Ares, Rheometric Scientific, Piscataway, NJ, USA) was used for the rheological measurements. The viscosity was measured in steady-state mode using the parallel plate geometry (radius 25 mm); the range of shear rate was set from 0.5 to 100 s^−1^. The rheological experiments were carried out at different temperatures (from 20 °C to 40 °C) and repeated at least three times at each temperature.

The pure PEG 800 and the LS/PEG800 were studied from a thermal point of view. A DSC1 (Stare System, Mettler Toledo, Columbus, OH, USA) instrument was utilized to assess the thermal properties and the phase transition range for the PCM. In this analysis, each sample was subjected to a thermal cycle of heating from −10 °C to 100 °C and cooling from 100 °C to −10 °C; in both the heating and cooling scans a 10 °C/min rate was used. The calorimetric tests were always performed in an inert atmosphere (i.e., nitrogen, with a flow rate of 60 mL/min). Each sample consisted of an average of 10 to 20 mg of material and was sealed in an aluminum crucible. For each formulation, three samples were analyzed, and the results were averaged. These experiments also allowed confirmation of the most appropriate temperature to perform the impregnation of PEG into the porous stone. Thermal characterization was completed by thermogravimetric analysis (TGA) using a simultaneous thermal analyzer (STA 409 produced by NETZSCH, Selb, Germany). This technique was used to monitor the mass variation of the PEG 800 and its LS composite in response to a temperature increase. The results of these analyses provided information on the thermal degradation process and on the amount of PEG 800 actually absorbed by the Lecce stone granules during the vacuum impregnation process. The thermogravimetric analysis, performed in a nitrogen atmosphere on specimens weighing between 10 to 20 mg, was carried out from 25 °C–600 °C at a heating rate of 10 °C/min. The thermogravimetric test was repeated three times on specimens of PEG 800 and LS/PEG800 in order to check the repeatability of the results.

The leakage behavior of the PCM composite materials (LS/PEG800 and LS/PEG1000) was investigated, as this was previously reported as a weakness in the use of low molecular weight PEGs to produce PCM [36,37]. Since no standardized test is currently available to measure this characteristic, a procedure reported in [36] was employed. In accordance with this method, 5 g of LS/PEG800 granules were placed on a preweighed filter paper, which was then placed in an oven (Binder FD 240, Waltham, Massachusetts, US) at a temperature close to the melting temperature of PEG 800 (30–35 °C); this was selected by taking into account the melting peak closure, as previously measured by the DSC. At the end of the 90-min test, the LS/PEG800 granules were removed from the filter paper; the difference between the initial and final weights of the filter paper assesses any PEG leakage from the stone flakes. This experiment was repeated using a higher temperature (75–80 °C) [36]. The test was repeated 3 times for each temperature, and the results were averaged. The same test was also performed on the LS/PEG1000. The 5 g of LS/PEG1000 on the filter paper was inserted into the oven for 90 min. In this case, the temperature was close to the melting point of PEG 1000 (45–50 °C as suggested by DSC results). Finally, the same test was repeated three times for both materials using a higher temperature (75–80 °C).

Lastly, the characterization of the two mortars (compositions reported in Table 1) was performed in both fresh and hardened states.

In accordance with European Standard EN 1015-3 [59], the workability of both mortars was assessed using the flow table test. 

Following European Standard EN 1015-11 [60], the mechanical performance of the mortars was analyzed in both flexural and compressive mode. For each mortar, 3 prismatic specimens (40 × 40 × 160 mm^3^) were made by forming the fresh mortar in iron molds. Samples of mortar remained in the mold for 2 days and were then demolded and cured for an additional 26 days in a proper room at standard temperature (25 °C) and humidity (50%) levels (total cure time was 28 days). The flexural tests were conducted in a Lloyd dynamometer (LR50K Plus by Ametek Company) at a speed of 6 μm/s; the compressive tests were executed by using the same machine at a speed of 12 μm/s.

Finally, the thermal characteristics and latent heats of the aerial lime mortars, with and without LS/PEG800 composite, were assessed employing the same technique (i.e., the DSC as used to characterize PEG 800 and its composite with LS). A thermal cycle was performed on each 10 to 20 mg-mortar specimens under a nitrogen atmosphere; heating was from −10 °C to 80 °C and cooling from 80 °C to −10 °C. (A lower maximum temperature was selected once it was observed that both PEG 800 and its composite with LS completed their melting/crystallization processes well below 100 °C.) The same heating and cooling rate was employed (10 °C/min) in order to compare the DSC results with those previously obtained by analyzing PEG 800 and LS/PEG800. Three mortar specimens (with or without the PCM) were analyzed by calorimetric analysis, and the DSC results were averaged. 

## 3. Results and Discussion

### 3.1. Characterization of PEG 800 and Its Form-Stable PCM

**Figure 1:**
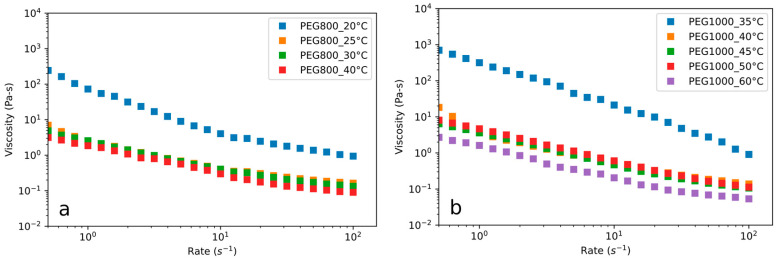
Viscosity vs. shear rate curves recorded at different temperatures of (**a**) PEG 800; (**b**) PEG 1000.

The most appropriate temperature to achieve a satisfactory inclusion of liquid PEG into the porous flakes of Lecce stone was identified by employing rheological measurements on PEG 800 at different temperatures (Figure 1a). For comparison, the results of the same test performed on PEG 1000 (as in [31]) are reported in Figure 1b. 

The viscosity of PEG 800 is characterized by a pseudo-plastic behavior, and it decreases with increasing temperature; the same qualitative behavior was already observed for PEG 1000. Starting from these results and taking into account that the melting process of pure PEG 800 is completed at 30 °C (as described in detail later in this paragraph), 40 °C was considered a suitable processing temperature, i.e., it would allow adequate impregnation of the Lecce stone granules with PEG 800.

**Figure 2:**
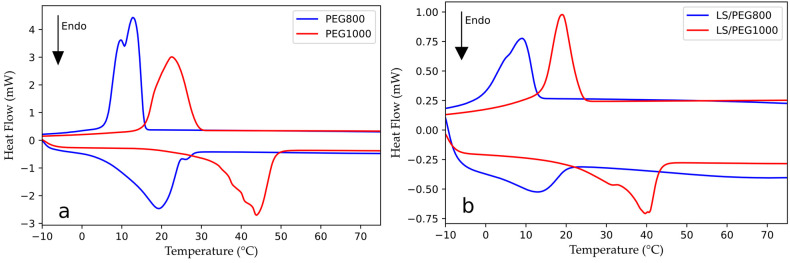
DSC curves recorded for (**a**) PEG 800 and PEG 1000 polymers; (**b**) LS/PEG800 and LS/PEG1000 composites.

To evaluate the LHTES properties of PEG 800 and of the LS/PEG800 composite, the measurement of their melting/crystallization temperatures along with latent heat capacities was performed in a DSC. The results of both the heating and cooling cycles are illustrated in Figure 2a for PEG 800 and 2b for its composite with Lecce stone. For comparison purposes, the results of PEG 1000 and the LS/PEG1000 composite are also reported in the same figures, respectively. In Table 3, the phase change temperatures and the related latent heat capacities are summarized.

As observable in Figure 2a and Table 3, on melting, PEG 800 exhibits two endothermic peaks: one centered at around 18 °C and a smaller one at 25 °C. Analogously, on cooling, two exothermic peaks are recorded for PEG 800: one at about 13 °C and another at 9 °C. The presence of two melting/crystallization peaks can be explained by the large molecular weight distribution of PEG 800 polymers, as has been previously reported in the literature [19,30,36,61]. Comparing the DSC results for the PEG 800 and PEG 1000 polymers, as expected, the prior displayed lower melting/crystallization temperatures than the PEG of greater molecular weight (around 43 °C and 24 °C on heating and cooling cycles, respectively). The melting/crystallization temperature ranges of LS/PEG 800 are closer to the typical temperatures of cold countries; both observations confirm that our choice could be effective in producing a PCM suitable not only for the colder seasons of the Mediterranean area, but also for geographic regions with colder climates, such as northern Europe. 

During the DSC experiments performed on the LS/PEG800 composite, an endothermic (melting) peak at around 13 °C and an exothermic (crystallization) peak at about 9 °C were measured. In LS/PEG composites, the melting and crystallization processes are usually found at lower temperatures, as observed for LS/PEG1000 and reported in our previous work [31]—this was taken into account in the selection of the most appropriate grade of PEG. 

Similar melting and crystallization enthalpies were calculated for PEG 800 (about 150 J/g). These results are roughly in line with what has been reported in the literature [19,30]. For the LS/PEG800 composite, where the (crystallizable) polymeric component is only a fraction of the whole system, much lower melting and crystallization enthalpies were recorded (around 28 J/g). These values are similar to those of previous studies [31], e.g., LS/PEG1000 as shown in Table 3. The latent heat collected during the phase change is in line with measurements from other studies on thermally-efficient, form-stable PCMs [19,22,30,36,62,63,64], proving it to be adequate for the intended purposes. The enthalpies recorded during the melting/crystallization processes of a form-stable PCM composed of a PEG polymer have generally been found to be slightly lower than those expected by normalizing the latent heat to the true PEG content. This has been explained by the difficulty of a PEG polymer to crystallize in the very small pores of an inert support (as compared to crystallization in isolation) [19,65,66].

To the best of our knowledge, only a few examples of PEG 800-based, form-stable PCM systems have been reported in the literature. In [30], PEG 800 was included in expanded graphite (EG). With a PEG 800 content of about 87% wt. (due to the high porosity of this support) the melting and crystallization enthalpies were 89.5 J/g and 80.6 J/g, respectively. However, including PEG 800 in the pores of expanded graphite is not a simple or inexpensive scheme. Additional work has developed chemical interactions between the PEG polymer and expanded graphite to improve the performance and stability of the PCM; however, these interactions can appreciably affect the crystallization process of PEG and the relative latent heats. In another work [19], different amounts of PEG 800 (up to 90% wt.) were included in an EG mesoporous structure. Latent heats for melting/crystallization ranged from 78 to 98 J/g—the higher the PEG content, the higher the enthalpy. It was underlined, however, that the mesoporous structure negatively affected the crystallization of PEG, causing a decrease in latent heat compared to pure PEG.

**Figure 3:**
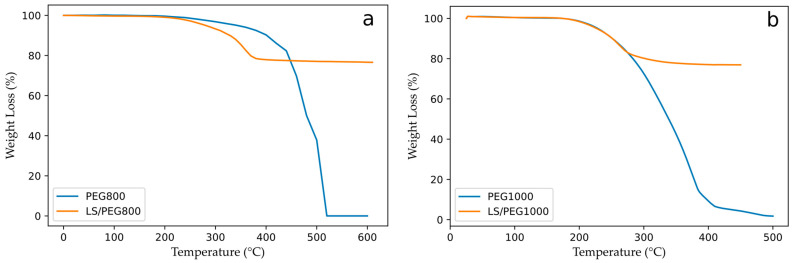
TGA experiments performed on (**a**) pure PEG 800 and LS/PEG800; (**b**) pure PEG 1000 and LS/PEG1000.

Thermogravimetric experiments were run to assess the thermal resistance and the degradation temperatures of pure PEG 800 and of its composite with Lecce stone, as these are important features for a PCM suitable for TES applications. The results are shown in Figure 3a and summarized in Table 4. The values for PEG 800 are nearly comparable with those of PEG 1000 (measured in [31] and reported for comparison purposes in Figure 3b and Table 4).

The degradation process in a non-oxidative (i.e., inert) atmosphere started at around 220 °C for both pure PEG 800 and its PCM-stone composite. On the other hand, the final temperature of the degradation process for LS/PEG800 was more than 100 °C lower than that of pure PEG 800. Nevertheless, the PEG 800-based PCM composite exhibits thermal stability and thermal reliability appropriate for the intended purposes, according to the literature [19,30,36]. 

TGA was also used to estimate the amount of the polymeric PEG component actually incorporated into the porous Lecce stone, which was calculated as the percentage of mass loss at the end of the TGA tests (around 390 °C) when the complete degradation of the polymer was achieved—this value is about 23%. This value is very close to the actual amount of PEG 1000 in LS/PEG1000 (as previously calculated using the same procedure); our previous studies have demonstrated this to be an efficient PCM for mortars [31,51,52].

Comparison between the TGA results for PEG 800 and its composite with those previously observed for PEG 1000 and LS/PEG1000 in [31] proved that they were comparable except for the final temperature of the degradation process, which was appreciably greater for both pure PEG 800 and its composite compared to PEG 1000 and LS/PEG1000. This could represent an advantage of the PEG-800-based PCM.

In conclusion, the thermal properties, as measured by DSC and TGA, as well as the amount of PEG 800 absorbed in the Lecce stone flakes, confirmed that the LS/PEG800 PCM offers suitable thermal behavior and an appropriate polymeric content. 

It is well known that the form-stable method is able to prevent leakage of the PCM from the matrix, as previously proven and explained in terms of a good affinity between the matrix and the polymer. This was demonstrated using matrices such as clay mineral [16], expanded graphite [19], expanded perlite [20,67], diatomite [35,62], and expanded vermiculite [21,63]. In previous work [31], a good affinity between LS and PEG 1000 was inferred from FTIR (Fourier-transform infrared spectroscopy) studies, which showed favorable physical interactions. As confirmation, a leakage test was carried out to assess if heating of the PEG 800 or PEG 1000 polymers caused them to come out of the stone grains; the results are reported in Table 5. 

**Figure 4:**
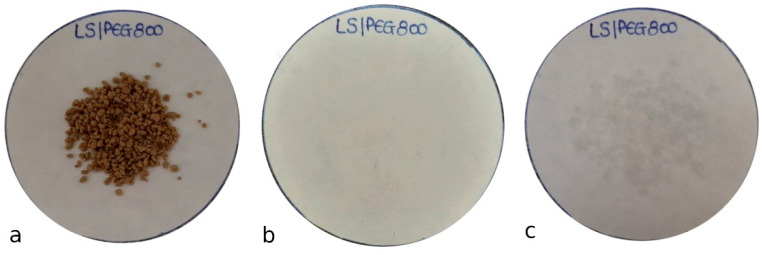
(**a**) LS/PEG800 PCM (5 g) placed on the filter paper, which had been previously weighed; (**b**) filter paper after the test performed at 30–35 °C; (**c**) filter paper after the test performed at 70–75 °C.

To conduct the test, the filter paper was weighed; then a known mass of composite material was placed on it (Figure 4a). Heating to a temperature close to the composite’s melting point (30–35 °C) produced no change in the weight of the filter paper; thus, no PEG was lost from the Lecce stone support. This was confirmed by the observation that the filter paper was unblemished after the test, as seen in Figure 4b. When a temperature much higher than the PEG800 melting point was used (75–80 °C), only a small change in the weight of the filter paper was recorded, corresponding to a loss of PEG of 0.3% by weight. Accordingly, a light stain on the filter paper is visible (Figure 4c). 

From the data reported in Table 5, similar conclusions can be drawn for LS/PEG1000: there was no loss of PEG 1000 from the LS granules around its melting point, and a small loss (0.4%) at a temperature much higher than its melting point.

The results indicate that no substantial PEG leakage occurred even during the test carried out at a temperature much greater than the melting point; thus, the support (Lecce stone granules) is capable of absorbing and retaining both PEG 800 and PEG 1000 polymers, giving rise to thermally stable PCMs. 

### 3.2. Characterization of Mortar Formulations

In Table 2, the aerial-lime-based mortar formulations created with or without the PEG 800-based PCM are reported. In our previous study [31], the same binder was used by adding a similar PCM, i.e., based on PEG 1000. It was found that a proper selection of mortar components would produce lime-based mortars with suitable workability; their mechanical properties, however, were not completely adequate. Based on the previous results, in this work the amount of binder was increased (from 800 kg/m^3^ to 1000 kg/m^3^) in order to enhance the mechanical properties. At the same time, the amount of superplasticizer was increased (from 15 kg/m^3^ to 20 kg/m^3^) in order to ensure a workability of the mortar when fresh.

In Table 6 reports the workability of the mortars. As they are within the range of 160–180 mm, both of the aerial-lime-based mortars exhibit suitable workability [31,51,68,69].

Table 7 presents the mechanical properties of the mortars. The aerial lime and PCM mortar (AL_LS/PEG800) experienced a decrease in both flexural and compressive strengths. The incorporation of the PCM leads to a reduction in flexural strength of more than half and an even greater decrease in compressive strength (around 70%). These results are perfectly in line with those recorded in our previous study on aerial lime mortars containing PCM based on PEG 1000 (LS/PEG1000) [31]. It is generally recognized that all mortars experience a reduction in mechanical properties upon the addition of a PCM composite [11], with aerial lime mortars typically being more affected [47].

According to EN 998-1 [56], both mortars fall in the lowest class: CSI. These results are in line with our previous investigation [31]. Based on their mechanical properties, therefore, the mortars produced in this study can be proposed for interior walls only. On the other hand, such lime-based mortars are especially recommended for indoor restoration of historic buildings, where it is mandatory to select materials similar to those originally used.

**Figure 5:**
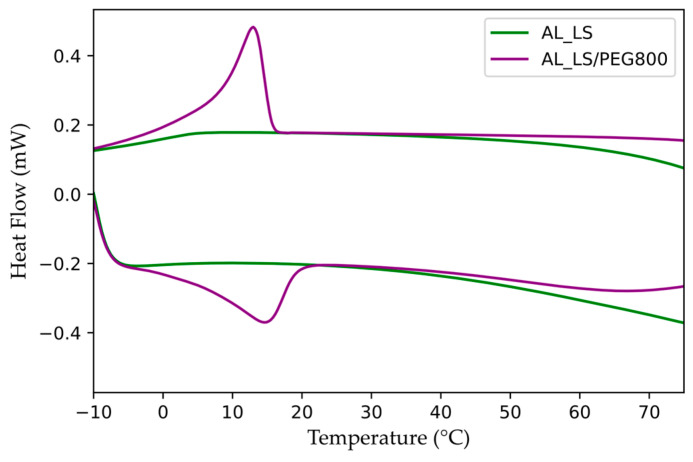
DSC curves recorded for aerial lime mortars with/without PEG800.

The thermal characterization of the aerial lime mortar containing the PEG 800-based PCM was performed using DSC. For comparison purposes, the same experiment was also performed on the AL_LS mortar (that not containing the polymeric component of the PCM). The DSC thermograms obtained for both mortar formulations are reported in Figure 5; the thermal behavior of the mortars can be compared with those displayed in Figure 2b for the LS/PEG 800 composite. The results of the calorimetric analysis performed on the mortar AL_LS/PEG800 are summarized in Table 8. 

From the observation of the curves reported in Figure 5, it is evident that the mortar not containing the polymeric component of the PCM (AL_LS) does not undergo any phase change, at least up to the temperatures analyzed, as expected. The mortar containing the PEG 800-based PCM, on the other hand, showed melting and crystallization peaks on heating and cooling cycles, respectively. The shapes of both peaks are similar to those for LS/PEG800 in isolation, as reported in Figure 2b. Comparing the DSC data calculated for the AL_LS/PEG800 mortar (Table 8) with those for the LS/PEG800 composite (Table 3), the melting and crystallization peaks appear to be only slightly shifted towards greater values. As expected, the latent heats measured during the melting/crystallization processes of AL_LS/PEG800 were found to be almost halved (but still easily measurable) of those of the LS/PEG800 composite. In line with other mortars with binders containing a PEG-based PCM [31], our results confirm that LS/PEG800 may be an effective system of energy storage if included in wall mortar for buildings located in Mediterranean countries during colder seasons or in regions located in continental climate. 

## 4. Conclusions

In this study, the first results of the “Development and analysis of mortars, based on different binders, containing a sustainable form-stable PCM able to improve the energy efficiency of the buildings located in different area” project are presented. Different grades of polyethylene glycol were selected in order to identify one with a melting-point range appropriate to the climate where the end-product will be used. Additionally, PEG polymers are eco-sustainable and are also used in biological and pharmaceutical applications. PEG 800 was selected due to its suitable range of melting/crystallization temperatures, which allowed the creation of PCMs suitable for lower temperature. As inert support for the PEG 800 to produce form-stable PCMs, fragments of porous Lecce stone were employed. The pieces of stone can be recovered as waste materials from the extraction and processing of stone in compliance with the principles of the circular economy. The preliminary calorimetric and rheological characterization of the PEG 800 allowed identification of the most suitable temperature to create the novel, sustainable PCM (LS/PEG800) through the form-stable method. A simple leakage test performed on PCM based on different PEGs (PEG800 and PEG1000) confirmed that these polymers remain inside the stone flakes even at temperatures comparable to or above their melting points. Calorimetric and thermo-gravimetric tests were performed on pure PEG 800 and on LS/PEG800 to measure the melting/crystallization enthalpies and temperature ranges and thermal degradation temperatures. By comparing these results with those previously recorded on PEG 1000 and its form-stable composite with Lecce stone (LS/PEG1000), it was possible to confirm the suitability of the PEG 800-based PCM for lower-temperature applications; the thermal resistance of LS/PEG800 was adequate for the intended purposes. Once the hypotheses that led to the choice of PEG 800 to create a form-stable PCM were confirmed, we employed it as aggregate for an aerial-lime-based mortar with the aim of producing an appropriate formulation with adequate physical properties (workability and mechanical performance). The same mortar containing the LS granules as aggregate was created for comparison. The aerial lime mortar containing the PEG 800-based PCM displayed adequate workability in its fresh state. Conversely, decreased mechanical (both flexural and compressive) properties were recorded for the aerial lime mortar containing LS/PEG800. Nevertheless, such new mortars can be used for interior walls, falling in the lowest class reported in EN 998-1. The LHTES properties measured on the aerial lime mortar containing LS/PEG800 composite confirmed that this PCM can be used to create mortar with the appropriate range of phase-change temperatures. Future research will focus on the production of other mortar formulations containing the form-stable PEG 800-based PCM in order to identify binders capable of maintaining good mechanical properties even in the presence of PCM. The durability of mortars containing the PEG 800-based PCMs with various binders will also be analyzed.

## Figures and Tables

**Table 1:** List of materials used in this work and description of their main properties.

Materials	Density	Supplied by	Main Properties
Lecce Stone (LS)	2957 kg/m^3^	L’essenza della Pietra, Taviano (Lecce), Italy	Stone with a high open porosity; available as waste material.
Polyethylene Glycol 800 (PEG800)	1200 kg/m^3^	Wuhan Fortuna Chemical, Wuhan, China	Low toxicity, low flammability; appropriate characteristic temperatures.
Aerial Lime (AL)	2450 kg/m^3^	Lhoist, Valverde, Portugal	Binder suitable for restoration and conservation of architectural heritage and historic buildings.
Polyacrylate (MasterGlenium SKY 627)	1050 kg/m^3^	BASF, Porto, Portugal	Superplasticizer used to minimize the quantity of water required by the mortar system.

**Table 2:** Mortar compositions (reported as kg/m^3^ of produced mortar).

Aggregates							
System	Binder/ Content	LS	PEG 800 Content	SP	Water Saturation ^1^	Water	Water/ Binder
AL_LS	Aerial Lime/1000	668	0	20	168	347	0.35
AL_LS/PEG800	Aerial Lime/1000	979	225	20	0	310	0.31

^1^ Water saturation is the water used to saturate the LS aggregate. This procedure is necessary because the LS aggregate has a high porosity—water saturation prevents the aggregate from absorbing the water required to produce the mortar. In the case of the LS/PEG composite, the saturation procedure is not necessary because the pores of LS are already saturated by PEG.

**Table 3:** LHTES properties measured in DSC on PEG 800, PEG 1000 and their PCM-stone composites, LS/PEG800 and LS/PEG1000: Onset and Endset, initial and final temperatures of the thermal process, respectively; Tm and Tc, peak temperatures of the melting and crystallization processes, respectively, recorded during heating/cooling stages; ∆H, enthalpy measured during heating/cooling stages. DSC data of PEG 1000 and LS/PEG1000 have been measured in [31].

		SAMPLES			
		PEG 1000	PEG 800	LS/PEG1000	LS/PEG800
**H**	Onset (°C)	35.1 ± 1.7	6.4 ± 2.3	31.7 ± 1.2	−5.3 ± 0.6
**E** **A**	Tm (°C)	42.8 ± 1.1	1st: 18.3 ± 0.9 2nd: 25.5 ± 1.1	39.3 ± 0.7	12.7 ± 1.4
**T**	Endset (°C)	55.2 ± 2.6	30.5 ± 1.9	47.7 ± 2.4	25.4 ± 2.2
**I** **N** **G**	ΔH (J/g)	129.3 ± 1.2	150.9 ± 10.3	27.7 ± 0.9	28.3 ± 3.4
**C**	Onset (°C)	28.9 ± 1.1	17.3 ± 1.1	23.5 ± 0.7	12.4 ± 0.8
**O** **O**	Tc (°C)	23.6 ± 1.2	1st: 13.3 ± 0.9 2nd: 9.1 ± 1.1	19.4 ± 0.9	9.3 ± 0.9
**L**	Endset (°C)	14.2 ± 0.7	−4.8 ± 0.9	9.3 ± 0.5	−8.2 ± 0.4
**I** **N** **G**	ΔH (J/g)	129.8 ± 0.8	151.4 ± 11.5	26.2 ± 1.1	28.1 ± 0.9

**Table 4:** Results of TGA experiments performed on PEG 800 and PEG 1000 and their PCM-stone composites. TGA data of PEG 1000 and LS/PEG1000 have been measured in [31]. (Onset and Endset represent the initial and final temperatures of the degradation process, respectively).

Sample	Onset (°C)	Endset (°C)	Residual (%)	Amount of PEG (%)
PEG 800	222.6 ± 2.1	520.6 ± 2.7	0 ± 0.0	100 ± 0.0
PEG 1000	220.2 ± 1.2	401.1 ± 1.1	1.7 ± 0.5	98.3 ± 0.5
LS/PEG800	219.2 ± 1.7	384.3 ± 1.8	76.6 ± 0.9	23.4 ± 1.3
LS/PEG1000	219.1 ± 1.2	292.6 ± 1.2	77.0 ± 1.0	23.0 ± 1.4

**Table 5:** Results from leakage tests performed on the composite materials LS/PEG800 and LS/PEG1000 at different temperatures: ΔWeight_paper_ is the difference in weight of the filter paper before and after the permanence of the LS/PEG granules in oven; Δ_LS/PEG_ is the decrease in weight (in percentage) of the LS/PEG granules at the end of the leakage test.

Sample	Temperature (°C)	ΔWeight_paper_ (mg)	Δ_LS/PEG_ (%)
LS/PEG800	30–35	0	0.0
	75–80	13	−0.3
LS/PEG1000	45–50	0	0.0
	75–80	20	−0.4

**Table 6:** Workability of the mortars whose compositions are reported in Table 2.

System	Workability (mm)
AL_LS	178
AL_LS/PEG800	160

**Table 7:** Mechanical performance measured on aerial lime mortars with or without LS/PEG800 PCM. Classification of mortars according to EN 998-1:2010 is also reported.

Sample	Flexural Strength (N/mm^2^)	Compressive Strength (N/mm^2^)	Classification EN 998-1:2010 [56]
AL_LS	0.63 ± 0.19	1.47 ± 0.16	CSI
AL_LS/PEG800	0.28 ± 0.12	0.44 ± 0.01	CSI

**Table 8:** LHTES properties measured in DSC on the aerial lime mortar containing LS/PEG800 composite: Onset and Endset are initial and final temperatures of the thermal process, respectively; Tm and Tc are the peak temperatures of the melting and crystallization processes, respectively, recorded during heating/cooling stages; ∆H is the enthalpy measured during heating/cooling stages.

	Heating				Cooling			
Sample	Onset (°C)	Tm (°C)	Endset (°C)	∆H (J/g)	Onset (°C)	Tc (°C)	Endset (°C)	∆H (J/g)
AL_LS/PEG800	3.3 ± 1.9	15.0 ± 1.0	24.1 ± 1.2	11.8 ± 0.4	17.7 ± 1.5	13.1 ± 1.1	−6.4 ± 0.9	12.5 ± 1.0

## Data Availability

The study does not report any data, please exclude the statement.
